# Trunnionosis After Total Hip Arthroplasty: A Review of the Etiology, Diagnosis, and Management

**DOI:** 10.7759/cureus.78037

**Published:** 2025-01-26

**Authors:** Muhammad Bin A Hamid, Zubair Younis, Mir Shahid-Ul Islam, Ahmed Hamed, Andalib Kashani, Muhammad Mannan, Rudra M Prabhu, Nayan Shrivastava, Ariz Raza, Nadia Rashid

**Affiliations:** 1 Trauma and Orthopaedics, University Hospitals Birmingham NHS Foundation Trust, Birmingham, GBR; 2 Trauma and Orthopaedics, The Royal Wolverhampton NHS Trust, Wolverhampton, GBR; 3 Trauma and Orthopaedics, Venkateshwara Institute of Medical Sciences, Gajraula, IND; 4 Orthopaedics, Seth Gordhandas Sunderdas (GS) Medical College and King Edward Memorial (KEM) Hospital, Mumbai, IND; 5 Pathology, Government Medical College, Srinagar, Srinagar, IND

**Keywords:** metallurgy, revision hip arthroplasty, total hip arthroplasty complications, total hip athroplasty, trunnionosis

## Abstract

Trunnionosis implies wear and corrosion at the head-neck junction of the femoral component in a total hip replacement, leading to the release of metal ions and particulate debris. It has become a recognized complication following total hip arthroplasty (THA), particularly in modular implant designs. These wear processes can result in adverse local tissue reactions (ALTRs), implant loosening, and systemic effects in severe cases. Factors contributing to trunnionosis include implant design, patient anatomy, activity level, and surgical technique, all of which influence the degree of mechanical wear and corrosion at the taper interface. Clinical presentation is variable, ranging from localized pain and limp to systemic symptoms of metal hypersensitivity or toxicity. Diagnosis requires a comprehensive approach, integrating clinical evaluation, imaging studies such as magnetic resonance imaging (MRI), serum metal ion levels, and synovial fluid analysis to differentiate trunnionosis from infection and other causes of implant failure. Management strategies focus on revision surgery, involving debridement of ALTRs, exchange of the femoral head, and addressing implant instability or loosening. As the use of modular implants continues to rise, understanding the etiology, diagnosis, and management of trunnionosis is essential to improving outcomes for patients undergoing THA.

## Introduction and background

Modularity has been a boon in the practice of total hip arthroplasty (THA) ever since its widespread adoption. This has made balancing leg length and offset much easier during hip arthroplasties [1]. It has, however, also been implicated in causing the phenomenon of trunnionosis, which is defined as wear at the femoral head-neck junction [2]. This is thought to be the reason for up to 3% of all THA revision procedures [3]. This corrosion and wear at the head-neck junction lead to the local release of cobalt-chromium ions, resulting in slow and progressive destruction of surrounding tissue [4]. There are several reasons that have been proposed for the same, but the underlying pathogenesis remains poorly understood. This review aims to delve into the etiology, pathogenesis, diagnosis, and management of trunnionosis after THA.

## Review

Etiology and pathogenesis

Mechanical Factors

Micromotion and fretting wear: Implant modularity has provided the modern arthroplasty surgeon with immense flexibility. It is one of the primary design elements of any total hip implant. The modular connections involve a male/female junction between a tapered shank and socket, connected via interlocking ridges [5]. Taper junctions are designed to self-lock and withstand considerable tensile, compressive, and rotational forces [6]. However, micromotion still occurs between the articulating surfaces, induced by cyclic loading. Most metal alloys used in orthopedic surgeries are subject to the formation of a passive film of metal oxides, to prevent further oxidation from taking place [5]. This micromotion causes the oxide layers to fracture, exposing the metal underneath. These cycles of oxidation and reduction contribute to the development of oxidation currents, generate wear particles, and facilitate the onset of corrosion. Also, large-diameter femoral heads, while postulated to decrease component instability, also impart greater torsional forces at the head-neck junction and contribute to mechanical wear [2]. It has been shown in finite element analysis models that stress in the femoral head-neck area increases with larger head diameters [7]. Along similar lines, shorter trunnion lengths, in effect, lead to the base of the trunnion within the femoral head, and this increases the likelihood of edge loading [8]. Trunnion diameters have also been hypothesized to increase the likelihood of micromotion at the head-neck interface. Research for this is, however, lacking, and there are conflicting reports supporting either assumption.

Micromotion at the interface of two similar metals results in disruption of the passivation layer and causes fretting corrosion [9]. This can be minimized by reducing the occurrence of micromotion at the head-neck taper. Obtaining a strong and stable press-fit is crucial to obtaining head-neck taper stability [10,11]. Micromotions of up to 5-12 µm are linked to fretting corrosion. Time spent in the body (an indirect marker for exposure to repetitive loads) is also linked to the degree of corrosion [12,13]. Obtaining a press-fit implies a stable taper connection, which is much less susceptible to micromotions and has a higher pull-off or fixation force. This is defined as the force required to remove the head from the taper and is a direct measure of taper stability.

Summarizing this, larger femoral heads and shorter trunnion lengths might accelerate the occurrence of trunnionosis.

Corrosion

In the THA femoral component, the head-neck connection is either metal-to-metal or metal-to-ceramic. The ‘female’ head is a tapered bore, and the ‘male’ stem is a tapered cone designed to fit into each other [14]. The cones and the head are often made of dissimilar alloys, with the cone typically being a cobalt-chromium-molybdenum alloy or titanium alloy, and the heads made of cobalt alloy, alumina ceramic, or zirconia ceramic [15-17]. The interface between dissimilar metals has been known to contribute to galvanic corrosion by the creation of a galvanic couple [9]. Relative motion within the crevice accelerates crevice corrosion in combination with local O2 depletion [18]. A process involving both fretting as well as crevice corrosion, as occurs in trunnionosis, is called mechanically-assisted crevice corrosion (MACC) [19]. This leads to elevated metal ions in synovial fluid as well as in serum and may lead to adverse local tissue reactions (ALTRs) [20]. ALTRs involve lymphocytic and macrophage infiltrates produced in response to particulate corrosion products, causing synovitis, osteolysis, and contributing to implant loosening. Long-term MACC could also ultimately lead to gross trunnion failure [21].

Patient-Related Factors

Body mass index (BMI): Higher body weight results in greater mechanical loads on the THA. This elevated load amplifies micromotion at the head-neck interface, leading to increased fretting and corrosion. Studies have shown that each 10 kg increase in body weight can cause a 1 µm increase in micromotion at this junction. This augmented mechanical stress, due to greater body weight, accelerates wear, contributing to the development of trunnionosis [22].

Activity level: In their research on realistic load testing for hip implants, Bergmann et al. have demonstrated that actual prosthetic loads are often several times the patient’s body weight, with impact activities increasing the prosthetic load manifold [23]. These loads, in turn, increase micromotion at the taper interface, thereby accelerating wear.

Anatomical factors: Patients with a high femoral neck offset often need longer heads to reproduce the same. This longer lever arm creates increased micromotion within the taper junction, and leads to a higher risk of corrosion [11].

Surgeon-Related Factors

Some of the factors that contribute to micromotion are under the direct control of the surgeon. Studies have shown that an impaction force of at least 4000 Newtons (N) is necessary to ensure proper seating of the prosthesis. This can be reached with a firm blow using a 500-gram hammer [24]. The impact with the highest energy controls the impaction force, and not the number of blows, as the effect is not cumulative [6,25]. It has been recommended that two blows be used for obtaining optimal impaction. The first blow is the alignment blow, and the second is the definitive impaction blow [26]. Attempts should be made to maintain good alignment between the impactor and the neck of the femoral stem. Maximum stability is achieved with impacts in the longitudinal axis of the taper junction. Off-axis impaction (even up to 10°) leads to reduced stability at the neck-stem interface [27].

Characteristics of impactors used also influence the final head-neck fixation force. Plastic impactors do not cause damage to the head but reduce fixation force compared to metallic ones. However, with ceramic heads increasingly used nowadays, that trade-off is unavoidable. Direct blows to the head must never be done to avoid point loading, and impactors with rubber tips should also be avoided due to inadequate stiffness [28].

Also, fat or fluid left on the trunnion at the time of assembly has a negative effect on fixation force [26,29,30]. It is crucial that the taper is cleaned and dried before assembly, and also to make sure that the bore is clean and dry as well. Cleaning the bore is difficult, and all attempts should be made to ensure that no fluid enters the bore during assembly [31]. Contamination due to fluid or fat at this junction reduces fixation force and increases the risk of micromotion and corrosion, even increasing the fracture risk for ceramic heads [32].

Diagnosis

Patients with trunnionosis can present with a range of symptoms, most commonly with pain and limp. These symptoms are non-specific; therefore, a wide differential must be considered when coming across such presentations [2,8,33]. In some cases, they can present with a rash as well, presumed to be induced by elevated metal ions and hypersensitivity [34,35]. Some cases can present with only a rash (localized/generalized), without pain. Some presentations can mimic iliopsoas tendinitis or trochanteric bursitis, or even mimic an apparent periprosthetic joint infection (PJI), necessitating the need for aspiration [35]. Patients with ALTRs to metal debris may present with a lump, or an abductor lurch, secondary to necrosis induced by the local tissue reaction [36].

All patients must be evaluated using plain radiographs of the hip. X-rays may show evidence of component loosening, calcar resorption, or osteolytic lesions. Cross-sectional imaging using ultrasound, computed tomography (CT) scans, or magnetic resonance imaging (MRI) may be considered. Metal artifact reduction sequence (MARS) MRI scans may show fluid, and the presence of increased synovial fluid, synovitis, or the presence of a pseudotumor around the involved hip [37,38].

All patients must also have erythrocyte sedimentation rate and C-reactive protein levels checked to screen for a PJI. Cobalt-chromium levels must be requested as well. There is evidence to suggest that a serum cobalt level >1 ng/mL or a serum chromium level >0.35 ng/mL in patients with a metal-on-polyethylene total hip replacement is abnormal, at any length of follow-up from index surgery [39]. There is also evidence to suggest that a differential elevation of cobalt, more than chromium, is often seen in trunnionosis. A serum cobalt-to-chromium ratio of >1.4 has been reported to have 93% sensitivity and 70% specificity for trunnion corrosion [40].

Often, the presentation in trunnionosis can mimic a PJI, and many of them might undergo a hip joint aspiration. Routine testing of joint aspirate for white cells and cultures should rule out infection; however, PJI and trunnion corrosion may co-exist. Also, corrosion products lead to inaccuracies in automated white cell counts; hence, a manual count should be requested in these scenarios [41].

Treatment

**Figure 1:**
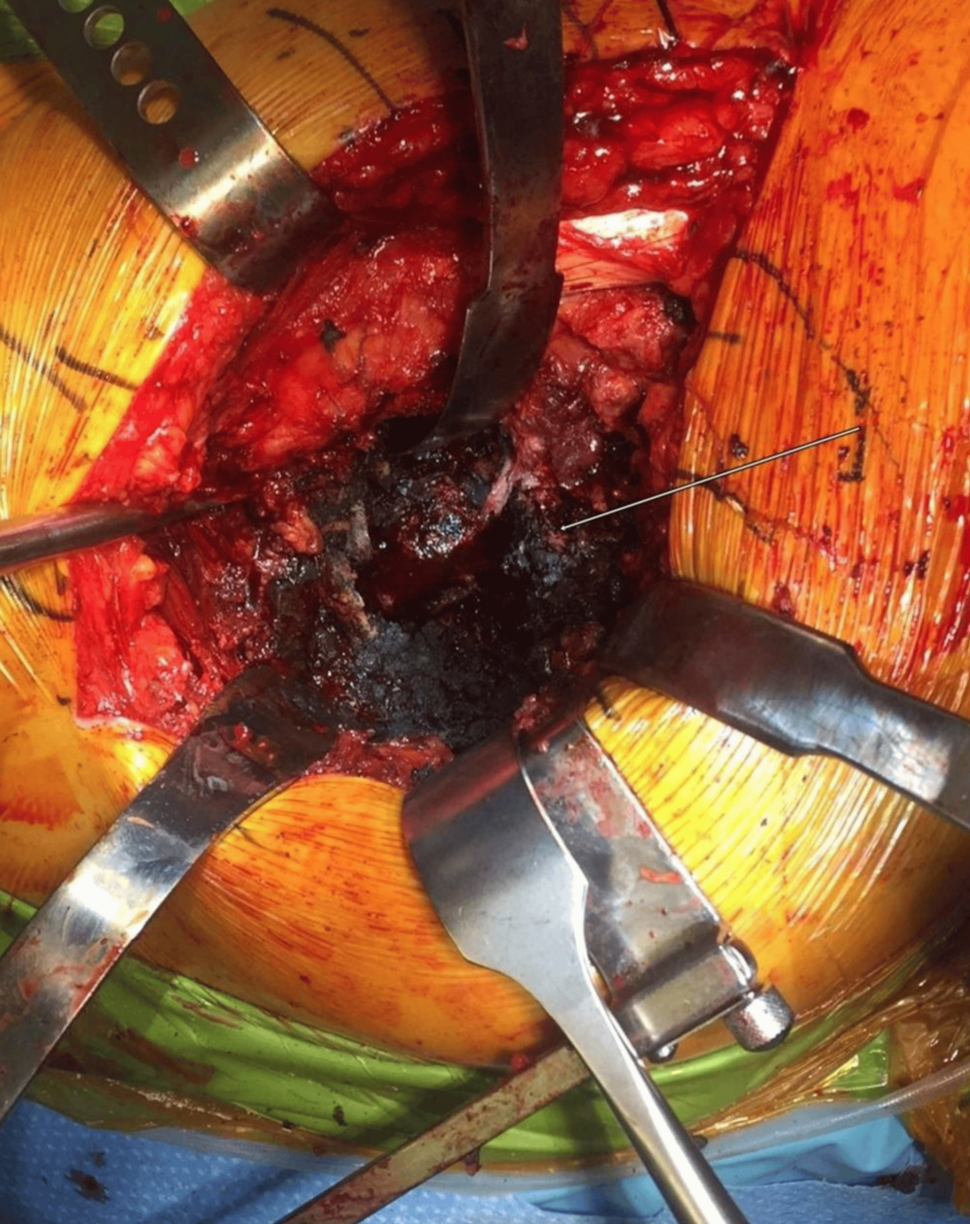
Intra-operative photograph of tissue staining from metallic corrosion debris. Image credit: Reproduced from [4]

There is no role for non-operative treatment in the management of symptomatic trunnion corrosion. Revision THA is often performed in patients with symptomatic corrosion, along with blood work and imaging suggestive of the same [42]. Most surgeons advocate debridement of synovial tissue, excision of pseudotumor, and removal of the offending femoral head (cobalt-chromium). Loose components must be revised [42]. As the morbidity of revising femoral stems is high, well-fixed femoral stems with no evidence of severe corrosion may be retained [8,43]. The femoral head is usually exchanged with a ceramic head with a titanium sleeve. The acetabular liner is exchanged concurrently as well. Custom titanium adapters can be used to match odd or uncommon taper geometry [44]. Debridement consists of sharp excision of all metal-induced ALTRs and pseudotumor from around the affected hip. Although not necessary, clinical photography of the affected soft tissues is often desirable (Figure 1) [4].

**Figure 2:**
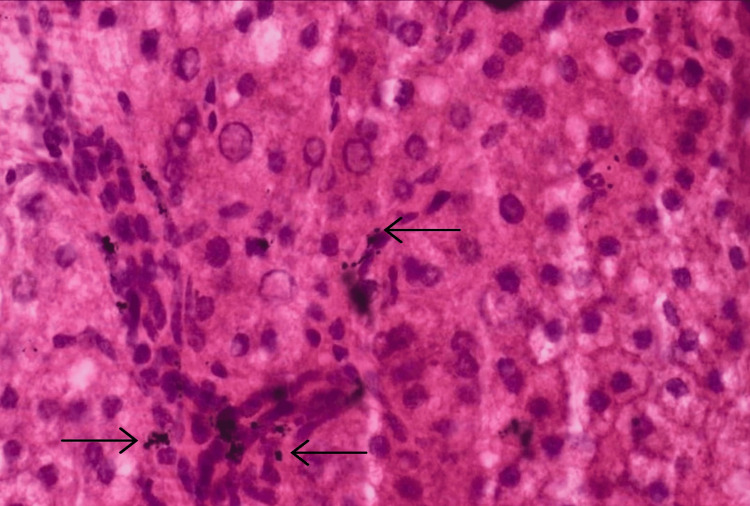
Hematoxylin and eosin-stained soft tissue at 100x showing metallic black particle debris in connective tissue (arrows). Image credit: Reproduced from [45]

Multiple fluid, soft tissue, and bone specimens must be sent for microbiological and histopathological analyses for infection and ALTRs. In symptomatic trunnion corrosion, histology would reveal dark, pigmented metal particle deposits within fibroconnective tissue (Figure 2) [45].

The incidence of post-operative complications is high after revision THAs are performed for trunnion corrosion. The most frequently reported complication following revision surgery is recurrent instability [46-48]. A study by Cooper et al. evaluated a total of 13 patients revised for trunnionosis with ALTRs. Out of the 13 patients, two had recurrent instability necessitating re-revision. One had sciatic nerve palsy, and one had a deep infection [46]. Plummer et al. reported a series of 27 patients revised for similar reasons. A total of 23 were revised with a ceramic head with titanium sleeve, while four had a new cobalt-chromium head. Of the 23 patients with a ceramic head, two patients had recurrent instability, one had a deep infection, and one had a peroneal nerve palsy. Of the four patients with cobalt-chromium heads, one developed a recurrence of ALTRs and required revision to a ceramic head with a titanium sleeve [48]. This might be reason enough to use a constrained liner or dual-mobility cup in patients with severe destruction of hip anatomy secondary to corrosion [42]. Similarly, Lash et al. reported major complications in six out of 10 patients operated for ALTRs. Two of these patients had recurrent dislocations. McGrory and Jorgensen also reported complications in seven out of 27 revisions performed for MACC after metal-on-polyethylene THA. Six of those had recurrent dislocations [49]. For this reason, it has been suggested to use a constrained liner or a dual-mobility component in cases where there is observed destruction of hip anatomy in order to reduce the risk of recurrent instability [42]. A large retrospective study of a specific type of metal-on-polyethylene THA evaluated 65 hips revised for MACC-induced trunnionosis. At 3.2 years following revision surgery, they found an overall major complication rate of 11.6% and a re-operation rate of 5.8% [50].

## Conclusions

Trunnion corrosion in modern metal-on-polyethylene THA is multi-factorial and can appear with myriad presentations. Early recognition can prevent the morbidity associated with symptomatic trunnionosis and also limit the extent of trunnion corrosion if revised early. A systematic approach, with clinical examination, radiology, and blood work given due consideration, would help arrive at the diagnosis and guide treatment. Delayed presentations may reveal destroyed hip anatomy upon revision, necessitating extensive surgery and increasing morbidity.
